# An evaluation of the major factors influencing the removal of copper ions using the egg shell (*Dromaius novaehollandiae*): chitosan (*Agaricus bisporus*) composite

**DOI:** 10.1007/s13205-016-0381-2

**Published:** 2016-02-23

**Authors:** R. K. Anantha, S. Kota

**Affiliations:** 1 Centre for Biotechnology, Acharya Nagarjuna University, Nagarjuna Nagar, Guntur, 522 510 Andhra Pradesh India; 2 Department of Biotechnology, Bapatla Engineering College, Bapatla, 522 101 Andhra Pradesh India; 3 Department of Biotechnology, RVR & JC College of Engineering, Guntur, 522 019 Andhra Pradesh India

**Keywords:** Adsorption, *Dromaius novaehollandiae* eggshells (DNES), Chitosan (CH), DNES–CH composite, Full factorial design, Copper

## Abstract

Rapid industrialisation, technological development, urbanization and increase in population in the recent past coupled with unplanned and unscientific disposal methods led to increased heavy metal levels in water. Realizing the need for development of eco-friendly and cost effective methods, the present investigation was done for the adsorptive removal of copper from aqueous solutions with *Dromaius novaehollandiae* eggshell and chitosan composite. By one variable at a time method, the optimum contact time was found to be 60 min with an adsorbent dosage of 8 g/L at pH 6, initial adsorbate concentration of 20 mg/L and temperature 30 °C. The equilibrium data followed Langmuir and Freundlich isotherm models and pseudo second-order kinetics. The equilibrium adsorption capacity determined from Langmuir isotherm was 48.3 mg/g. From the Van’t Hoff equation, thermodynamic parameters such as enthalpy (Δ*H*°), entropy (Δ*S*°) and Gibb’s free energy (Δ*G*°) were calculated and inferred that the process was spontaneous, irreversible and endothermic. To know the cumulative effects of operating parameters, a three level full factorial design of Response Surface Methodology (RSM) was applied and the suggested optimum conditions were 7.90 g/L of adsorbent dosage, 20.2651 mg/L of initial adsorbate concentration and 5.9 pH. Maximum percentage of copper adsorption attained was 95.25 % (19.05 mg/L) and the residual concentration of the metal after sorption corresponded to 0.95 mg/L, which is below the permissible limits (1.3 mg/L) of copper in drinking water. The adsorbent was characterized before and after adsorption by SEM–EDS, FTIR and XRD. The FTIR analysis showed the involvement of carboxyl, hydroxyl and amino groups while XRD analysis revealed the predominantly amorphous nature of the composite post-adsorption and the peaks at 2*θ* angles characteristic for copper and copper oxide. The mechanisms involved in the adsorption of copper onto the adsorbent are chemisorption, complexation and ion exchange.

## Introduction

Imperfect industrial activities like mining, electroplating, printing, photography, etc. and non-uniform technological development are the major factors contaminating the environment with toxic substances including heavy metals. These metals pose a significant threat to the environment and the health of living organisms because of their toxicity due to accumulation in living tissues. Metal cleaning and plating baths, pulp, paper board mills, printed circuit board production, wood pulp production, fertilizer industry, etc. are releasing copper into the environment. An ultra trace amount of copper is essential for living organisms but excess is detrimental. In human beings, ailments such as stomach upset and ulcers, liver and brain damage are caused by the excess concentrations of copper (Zhu et al. 2009). According to Safe Drinking Water act, copper has a permissible limit of 1.3 mg/L in drinking water (Johnson et al. 2002). Therefore removal of excess copper from the contaminated waters is the most important environmental issue of worldwide concern since fresh water is the basic need for biotic community. Also, depletion of copper sources could be avoided by recovery.

Many conventional methods have been developed for the removal of heavy metals from effluents such as sedimentation, ion exchange, filtration and membrane processes, electrochemical processes, chemical precipitation and solvent extraction. But these methods are inefficient when the concentrations of metals are low (below 100 ppm), and are also associated with certain disadvantages like high capital investment and operational costs, high sensitivity to operational conditions, significant energy consumption and production of large quantities of waste. To overcome these technical and economical barriers, development of ecofriendly, efficient and low-cost processes is of prime significance. In this aspect, adsorption is regarded as an innovative technology with the advantages of high efficiency and selectivity for adsorbing metals even when present in low concentrations, easy desorption of metals, recycling of the adsorbents and minimization of sludge generation.

During the last decade, several studies showed that microorganisms like bacteria (Arul Manikandan et al. 2014), fungi (Huang and Lin 2015; Kan et al. 2015) and algae (Ajjabai and Chouba 2009), agricultural wastes like *Sophora japonica* pods powder, coconut tree sawdust, sugarcane bagasse, water melon seed hulls and coir fiber (Amer et al. 2015; Putra et al. 2014; Gulbahar and Guzel 2013; Shukla and Shukla 2013), poultry wastes like shells/feathers (Ratna Kumari and Sobha 2012), *Catla catla* fish scales (Venkatesa Prabhu et al. 2012) and water snail/shrimp shell wastes (Mohanasrinivasan et al. 2014) could be used for bioremediation. Several synthetic polymer composites like activated carbon/chitosan, chitosan supported on porous glass beads, magnetic carboxymethyl chitosan, crosslinked alumina chitosan, Fe_3_O_4_/chitosan/polycomposite, crosslinked chitosan acrylonitrile copolymer and n-HAP/chitosan composite (Huang et al. 2014; Shen et al. 2013; Guijuan et al. 2012; Zhang et al. 2012; Ramya et al. 2011; Rajiv Gandhi et al. 2011), mesoporus and ligand composites (Awual and Hasan 2015; Awual 2015; Awual et al. 2013, 2014a, b, 2015; Espergham et al. 2011; Ghaedi et al. 2009) etc. are effective in the removal of copper.

Irrespective of the biosorbent material, the main governing factors in selecting a suitable adsorbent are sorption capability, abundant local availability, low cost and ecofriendly nature. In compliance with the said factors, the authors used the discarded egg shell wastes (ES) from the poultry farms of *Dromaius novaehollandiae* (Emu) and synthesized DNES–Chitosan (CH) composite as an adsorbent to remove copper from aqueous solutions. ES material, considered a waste, can in fact serve two important functions: one is its utilization in the adsorption of toxic metals there by cleaning up the polluted water and the other is the avoidance of its own disposal cost. The emerald green coloured emu eggshells (DNES) and the soft gel forming chitosan (2-acetamido-2-deoxy-β-d-glucose-*N* acetylglucosamine) processed from white mushroom, *Agaricus bisporus*, were combined in the ratio of 5:1 to get DNES–CH composite. In the present work, uptake of copper by DNES–CH composite was investigated with respect to vital parameters like contact time, pH, adsorbent dosage, initial metal concentration and temperature. Pseudo first order, pseudo second order and intra-particle diffusion kinetic models were used to describe the process kinetics. To analyze the adsorption equilibrium, three isotherm models viz. Langmuir, Freundlich and Temkin were used. Maximum sorption capability predicts the amount of the sorbent required for effective sorption and it was obtained from Langmuir isotherm. From the Van’t Hoff equation, thermodynamic parameters such as enthalpy change (Δ*H*°), entropy change (Δ*S*°) and Gibb’s free energy (Δ*G*°) were calculated. To study the cumulative and interactive effects and optimization of copper adsorption process, full factorial design of RSM was applied with three independent variables at three levels. To know the interactions between the adsorbent and the adsorbate, Scanning Electron Microscopy (SEM)–Energy Dispersive Spectroscopy (EDS), Fourier Transform Infra Red spectroscopy (FTIR) and X-Ray Diffraction (XRD) analyses were performed.

## Materials and methods

### Adsorbents

Egg Shells of *Dromaius novaehollandiae* (DNES) were collected from the poultry processing facilities of Bapatla, Andhra Pradesh, India. Shells were washed several times with deionized water to remove dirt particles. The eggshells were then ground to powder, sieved using British Standard Sieves (BSS) into particle size of 53 µm (chosen from different particle sizes tested) for use as sorbent without any pretreatment. Chitosan (prepared from *Agaricus bisporus*) was purchased from Sigma Aldrich (740179). DNES–CH composite was prepared by first dissolving CH and DNES separately in 10 and 2 % aqueous acetic acid respectively. Further eggshells were treated with 2 % NaHCO_3_ for neutralisation. Then the neutralized DNES solution was added to CH solution and the mixture was dropped into an alkaline coagulant solution (H_2_O:MeOH:NaOH = 5:4:1 w/w). The DNES–CH (5:1) composite thus obtained was washed with distilled water and stored for experimental use.

### Adsorbate

Stock solution was prepared by dissolving 3.798 g of Cu(NO_3_)_2_·3H_2_O in 250 mL of deionized water, diluted to 1 L in a volumetric flask with double distilled water procured from Millipore ELIX-10 unit. Test solutions were prepared by progressive dilution of stock solution of copper with double distilled water and the pH was adjusted to the desired value by using 0.1 N HNO_3_ or 0.1 N NaOH solutions. All chemicals used were of analytical grade.

### Adsorption experimental procedure

Adsorption was carried out in a batch process by contacting 300 mg of DNES-CH composite with 50 mL of 20 mg/L of copper solution. In the preliminary step, all adsorption experiments were conducted in 250 mL Erlenmayer flasks using one variable at a time. The flasks containing the adsorbate and the adsorbent were agitated on an orbital shaker (REMI make CIS-24BL) at 180 rpm and 30 °C temperature. Samples were taken at predetermined time intervals of 1, 5, 10, 15, 20, 25, 30, 40, 50, 60, 70, 80 and 90 min. For further experiments, contact time was adjusted to optimum. The effect of pH was studied in the range of 2–9. With optimum contact time and pH, metal solutions in the concentration range of 20–100 mg/L were used to assess the effect of initial copper ion concentration. Similarly, the adsorbent dose was varied from 150 to 450 mg and finally temperature was varied between 10 and 50 °C. Each of the experiments was repeated twice and the average values were obtained. At the end of each adsorption process, the adsorbate was filtered out through whatman filter paper and the residual metal concentration was determined by Atomic Absorption Spectrophotometer (Shimadzu make AA-6300) with copper hallow cathode lamp using air acetylene flame at a wavelength of 324.8 nm. The percentage removal was obtained by using the following expression:

1 $$ {\text{Percentage}}\;{\text{removal}}\; ( {\text{\% )}}\;{\text{of}}\;{\text{metal}}\; = \;\left[ {\left( {C_{\text{o}} - C_{\text{e}} } \right)/C_{\text{o}} } \right] \times 100 $$

where *C*_o_ is the initial concentration of stock sample (mg/L), *C*_e_ is the final concentration of stock sample after adsorption (mg/L).

### Adsorption kinetics, isotherms and thermodynamic studies

Kinetic models including pseudo-first-order, pseudo-second-order and intra-particle diffusion were used to determine the rate of adsorption of copper. The sorption equilibrium between the adsorbent and the metal ions was described by using Langmuir, Freundlich and Temkin models. For all the three models, the isotherm constants were obtained by non-linear regression methods. Thermodynamic parameters were calculated using Van’t Hoff equation.

### Experimental design and statistical analysis

Three level full factorial design of RSM was used to optimize the chosen design variables (Ghaedi et al. 2015; Srivastava et al. 2014) for the optimization of copper adsorption by DNES–CH composite. Experiments with three initial adsorbent dosages (*X*_1_) 7, 8 and 9 g/L, initial metal (adsorbate) concentrations (*X*_2_) 15, 20 and 25 mg/L and initial pH values (*X*_3_) 5, 6 and 7 were employed simultaneously according to the design. Experiments (28 runs) were conducted to describe the effects of all the three variables on adsorption. The coded values of the process parameters were determined by the equation

2 $$ x_{i} = \frac{{X_{i} - X_{\text{o}} }}{{\Delta X}} $$

where *x*_*i*_ is the coded value of the *i* ^th^ variable, *X*_*i*_ is the uncoded value of the *i* ^th^ test variable and *X*_o_ is the uncoded value of the *i* ^th^ test variable at the center point. The levels of the coded variables are given in Table 1. The behaviour of the experimental design is explained by the following polynomial second order equation

3 $$ Y = \beta_{0} + \mathop \sum \limits_{i = 1}^{k} \beta_{i} X_{i} + \mathop \sum \limits_{i = 1}^{k} \beta_{ii} X_{i}^{2} + \mathop \sum \limits_{i = 1}^{k - 1} \mathop \sum \limits_{j = 2}^{k} \beta_{ij} X_{i} X_{j} $$

Design expert 9 (Stat-Ease Inc., Minneapolis, MN, USA) software trial version was used for statistical analysis of the experimental results. Regression equation was solved using fmincon function of Matlab 2008 and with the optimum values obtained, surface plots were drawn.

**Table 1:** Full factorial design for adsorption of copper by DNES–CH composite

Run no.	Coded values			Unoded values			% Adsorption		Residual	% Error
	*X*_1_	*X*_2_	*X*_3_	*X*_1_	*X*_2_	*X*_3_	Actual	Predicted		
1	−1	−1	−1	7	15	5	69.24	68.36	0.88	1.27
2	0	−1	−1	8	15	5	88.26	90.67	−2.41	2.73
3	1	−1	−1	9	15	5	83.87	80.75	3.12	3.72
4	−1	0	−1	7	20	5	76.38	78.08	−1.67	2.18
5	0	0	−1	8	20	5	95.97	94.52	1.45	1.51
6	1	0	−1	9	20	5	74.85	78.77	−3.92	5.23
7	−1	1	−1	7	25	5	83.87	83.15	0.72	0.85
8	0	1	−1	8	25	5	93.63	93.79	−0.16	0.17
9	1	1	−1	9	25	5	74.18	72.20	1.98	2.66
10	−1	−1	0	7	15	6	71.68	72.18	−0.50	0.69
11	0	−1	0	8	15	6	91.19	92.40	−1.21	1.32
12	1	−1	0	9	15	6	81.43	80.39	1.04	1.27
13	−1	0	0	7	20	6	81.33	80.87	0.46	0.56
14	0	0	0	8	20	6	98.27	95.25	3.02	3.07
15	1	0	0	9	20	6	74.12	77.40	−3.28	4.42
16	−1	1	0	7	25	6	85.12	84.98	0.14	0.16
17	0	1	0	8	25	6	90.99	93.52	−2.53	2.78
18	1	1	0	9	25	6	69.66	69.83	−0.17	0.24
19	−1	−1	1	7	15	7	74.12	74.37	−0.25	0.33
20	0	−1	1	8	15	7	93.63	92.49	1.14	1.21
21	1	−1	1	9	15	7	76.56	78.39	−1.83	2.39
22	−1	0	1	7	20	7	81.47	82.06	−0.59	0.72
23	0	0	1	8	20	7	94.87	94.34	0.53	0.55
24	1	0	1	9	20	7	75.39	74.40	0.99	1.31
25	−1	1	1	7	25	7	85.97	85.16	0.81	0.94
26	0	1	1	8	25	7	88.75	91.61	−2.86	3.22
27	1	1	1	9	25	7	67.89	65.83	2.06	3.03
28	0	0	0	8	20	6	98.27	95.25	3.02	3.07

### Characterization of the biosorbent

To observe the surface changes of the adsorbent after sorption of the metal ions and in situ metal analysis, SEM (EVO 18 make Carl Zeiss) and EDS (Oxford instrument, Inca) analyses were employed respectively. To know the main functional groups present on the adsorbent and their interaction with the metal ions, Fourier transform Infrared (Bruker UK, ATR) analysis was performed. The adsorbent was also characterized by X-ray diffraction (XRD) technique using X-ray diffractometer with Cu Kα radiation (*λ* = 1.5406 Å). The measurement was in the scanning range of 5–100 at a scanning speed of >19.685 s^−1^.

## Results and discussion

### Effect of contact time

**Fig. 1:**
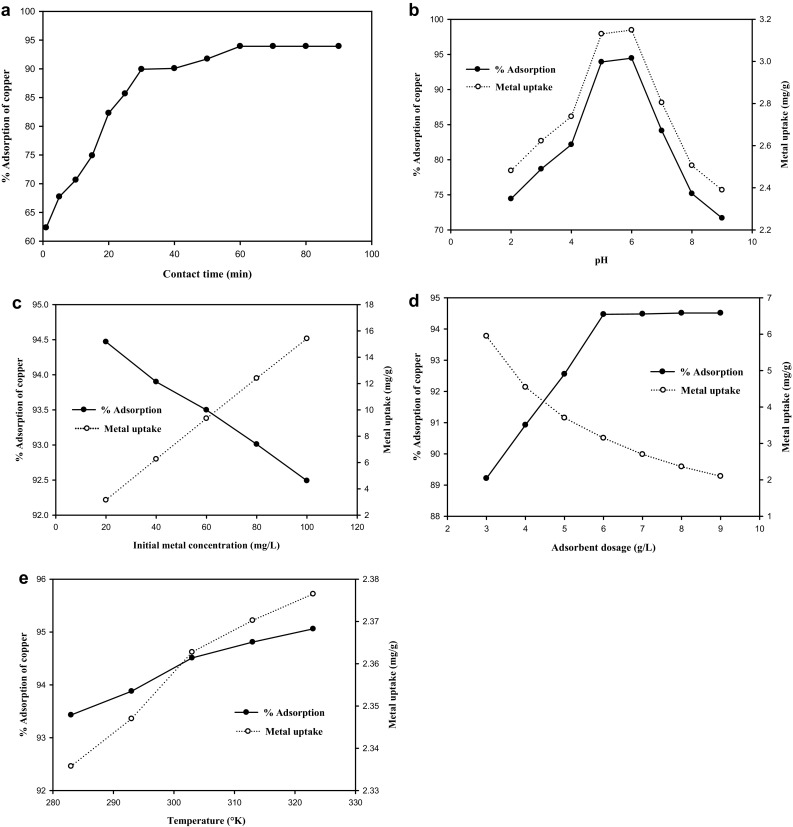
**a** Effect of contact time on % adsorption of copper. **b** Effect of pH on % adsorption and metal uptake of copper. **c** Effect of initial metal concentration on % adsorption and metal uptake of copper. **d** Effect of adsorbent dosage on % adsorption and metal uptake of copper. **e** Effect of temperature on % adsorption and metal uptake of copper

The results showed that the percentage removal of copper increased briskly up to 60 min reaching 93.925 % (Fig. 1a). Beyond 60 min, the % of adsorption remained the same indicating the attainment of equilibrium conditions. Equilibrium indicates utilization of the more readily available adsorbing sites especially the carboxyl and the amino groups present on the DNES–CH composite surface. Initial rates of sorption are faster due to the availability of adequate vacant surface binding sites and the vanderwaal forces of attraction between the adsorbate and the adsorbent. With the advancement of time, sorption becomes gradually tapered due to repulsive forces between the solute molecules of the solid and the bulk phases; consequently, the remaining vacant binding sites fail to bind with the metals (Srivastava et al. 2014). The reported contact time of 150 min for removal of copper by chick egg shells by packed-bed sorption (Nabil and Sameer 2009) is much higher than the optimum contact time of 60 min for the composite in the present study.

### Effect of pH on adsorption

The pH of the aqueous metal solution affects sorption through the adsorbent surface charge, the degree of ionization and speciation of the adsorbate as well. Maximum  % removal of copper was 94.47 % at pH 6 (Fig. 1b) and this is in agreement with the earlier research reports (Rafatullah et al. 2010; Chen et al. 2012). At pH < 5, the number of sites available for metal adsorption will be low as most of the functional groups are protonated and H_3_O^+^ ions compete with the metal for the adsorption sites on the adsorbent. At pH 6, the increase in adsorption could be attributed to the weak inhibitory effect of H_3_O^+^ ions. At pH above 6, the adsorption rates were unpredictable due to accumulation of metal ions on the adsorbent.

### Effect of initial metal ion concentration

The maximum percentage removal of copper observed was 94.47 % when the initial copper concentration was 20 mg/L (Fig. 1c). The plot suggests that the metal ion adsorption increased sharply in the beginning and then decreased slowly with further increase in the initial concentration. This can be attributed to the increase in adsorbate concentration to a fixed number of available active sites on the adsorbent (Malkoc 2006).

### Effect of adsorbent dosage

The experiments were carried out by varying the adsorbent loading from 3 to 9 g/L. The percentage removal of copper by DNES-CH composite at different adsorbent doses is presented in Fig. 1d. Experimental studies were carried out at 30 °C with an initial metal concentration of 20 mg/L at pH 6. The removal of copper increased rapidly from 89.215 to 94.51 % with an increase in the adsorbent dose from 3 to 8 g/L. As shown in the figure, the percentage removal increased initially with the increase of the adsorbent dose due to increased surface area of the adsorbent and the number of binding sites. For a fixed initial metal concentration, although the adsorbent dose was increased, there was no significant change in the adsorption after attaining the equilibrium (Witek-Krowiak et al. 2011; Moreno-Pirajan et al. 2011).

### Effect of temperature

Adsorption is expected to increase by an increase in temperature owing to the increase in the rate of diffusion of the adsorbate molecules across the external boundary layer and into the internal pores of the adsorbent particles. In this case, copper uptake marginally increased from 93.43 to 94.51 % for DNES–CH composite with increasing temperature from 10 to 30 °C indicating that the adsorption of copper on to the adsorbent is an endothermic process. With further rise in temperature beyond 30 °C, the increase in copper uptake was only marginal (Fig. 1e).

### Adsorption kinetics

The kinetics of adsorption of copper at different contact times was found out using the pseudo-first order, pseudo-second order, and intra-particle diffusion models. The pseudo-first order equation is

4 $$ \log \left( {q_{e} - q_{t} } \right) = \log \, q_{e} - K_{1} /2.303\left( t \right) $$

where *q*_*e*_ and *q*_*t*_ are the amount of metal adsorbed at equilibrium and at time *t*; *K*_1_ is the rate constant of pseudo-first order (min^−1^).

5 $$ {\text{The pseudo-second order equation is }}t/q_{t} = \, 1/K_{2} q_{\hbox{e} }^{2} + \, 1/q_{\hbox{e} } (t) $$

where *K*_2_ (min^−1^) is the pseudo-second order rate constant.

Intra-particle diffusion equation is

6 $$ qt \, = \, K_{\text{int}} t^{1/2} + C $$

where *K*_int_ is intra-particle diffusion rate constant (mg/g min^1/2^) and *C* is the boundary layer thickness.

**Fig. 2:**
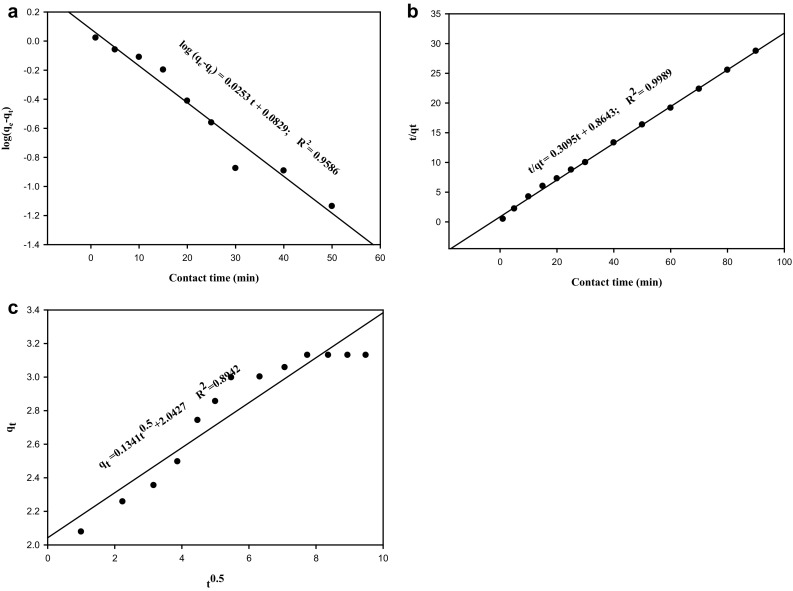
**a** Pseudo-first order kinetics for adsorption of copper using DNES–CH composite. **b** Pseudo-second order kinetics for adsorption of copper using DNES–CH composite. **c** Intra-particle diffusion kinetics for adsorption of copper using DNES–CH composite

The pseudo-first order, pseudo-second order and intra-particle diffusion kinetic plots are given in Fig. 2a–c respectively. In the pseudo-first order, the *K*_1_ and *q*_*e*_ values were 0.0582 (min^−1^) and 1.2103 (mg/g) respectively with a correlation coefficient (*R* ^2^) of 0.9586. In the pseudo-second order, the *K*_2_ and *q*_*e*_ values were 0.1104 (min^−1^) and 3.2362 (mg/g) respectively with a correlation coefficient (*R* ^2^) of 0.9989. The intra-particle diffusion, a plot of solute sorbed against square root of contact time, should normally yield a straight line passing through the origin but the line in the present case, did not pass through the origin and the values of *K*_int_ and *C* were 0.1341 (mg/g min^½^) and 2.0427 (mg/g) respectively with a correlation coefficient (*R* ^2^) of 0.8942. From the experiments, *q*_*e*_ obtained was 3.1308 mg/g which is suggestive that the adsorption of copper by DNES–CH composite could be by chemisorption, appropriately explained by the pseudo-second order model.

### Adsorption isotherm

The experimental data were tested and compared with the three isotherm models viz. Langmuir, Freundlich and Temkin. Langmuir isotherm is the most widely used model and the equation is given as

7 $$ \left( {c_{e} /q_{e} } \right) = 1/\left( {bq_{m} } \right) + c_{e} /q_{m} $$

**Fig. 3:**
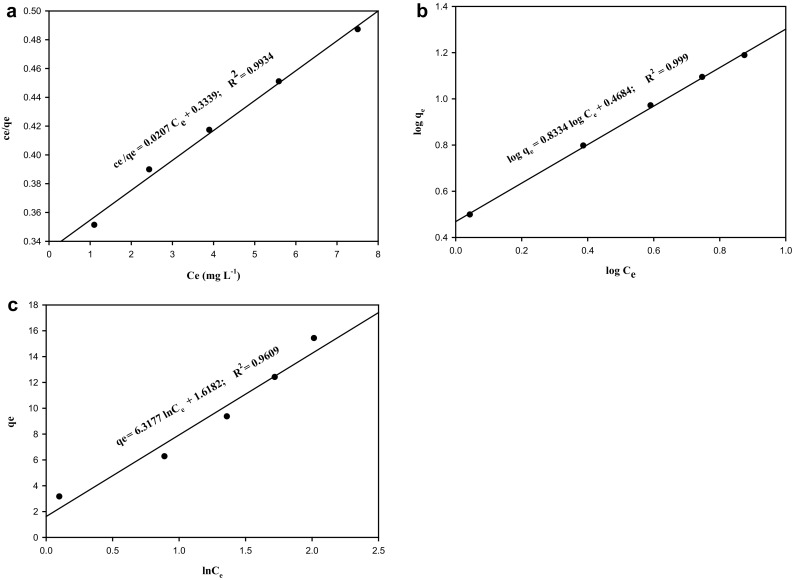
**a** Langmuir isotherm for adsorption of copper using DNES–CH composite. **b** Freundlich isotherm for adsorption of copper using DNES–CH composite. **c** Temkin isotherm for adsorption of copper using DNES–CH composite

The equation obtained for the Langmuir isotherm (Fig. 3a) for the current data was *c*_*e*_/*q*_*e*_ = 0.0207*c*_*e*_ + 0.3339 with a correlation coefficient of 0.9934 indicating the strong binding of copper ions on to the surface of the adsorbent. The maximum metal uptake (*q*_max_) obtained was 48.3 mg/g and the separation factor (*R*_*L*_) was 0.9358, indicating that the adsorption is favorable (0 < *R*_*L*_ < 1). Freundlich isotherm, applied in the cases of low and intermediate concentration ranges, is given by

8 $$ q_{e} = \, K_{f} C_{e}^{1/n} $$

where *K*_*f*_ and *n* are the adsorption capacity and intensity respectively. Freundlich equation linearized in logarithmic form as follows:

9 $$ \log \, q_{e} = \log K_{f} + n\log C_{e} $$

with the experimental data represented in Fig. 3b, the Freundlich equation obtained was log *q*_*e*_ = 0.8334 log *C*_*e*_ + 0.4684 with a correlation coefficient of 0.999. As the *n* value was 0.8334, which is in between 0 < *n* < 1, good adsorption of copper ions over the entire range of concentrations studied was inferred. The *K*_*f*_ value obtained was 2.9403 mg/g. Temkin isotherm equation describes the behavior of many adsorption systems on the heterogeneous surface. The linear form of the equation

10 $$ q_{e} = \left( {{\text{RT}}/b_{T} } \right)\ln \left( {A_{T} } \right) + \left( {{\text{RT}}/b_{T} } \right)\ln \left( {c_{e} } \right) $$

was used for analyzing the experimental data and presented in Fig. 3c. The equation obtained for copper adsorption was *q*_*e*_ = 6.3177 ln *c*_*e*_ + 1.6182 with a correlation coefficient of 0.9609.

The applicability of each model was evaluated using error analysis (*χ* ^2^). To find out the best-fit isotherm, the non-linear Chi square test statistic (*χ* ^2^)

11 $$ \chi^{2} = \varSigma \left( {q_{e,\exp } - q_{{e,{\text{cal}}}} } \right)^{2} /q_{{e,{\text{cal}}}} $$

where, *q*_*e*,exp_ *q*_*e*,cal_ are experimental and calculated adsorption capacity values respectively, was used (Ho and Ofomaja 2005).

The good agreement of the data acquired by each model needs that the *χ* ^2^ value is a smaller number while the non-applicability of the model has larger *χ* ^2^ value. The isothermal constants and *χ* ^2^ values are indicated in Table 2. With the use of the Chi square test (*χ* ^2^) characteristic and the regression correlation coefficient *R* ^2^, the best isothermal model was identified as the Langmuir followed by the Temkin and the Freundlich isothermal models.

**Table 2:** Isotherm constants of copper adsorption onto DNES–CH composite

Langmuir	Freundlich	Temkin
*q*_max_ = 48.3 mg/g	*n* = 0.8334	*A*_*T*_ = 1.2918
*R*_L_ = 0.9358	*K*_*f*_ = 2.9403 mg/g	*b*_*T*_ = 398.743
*R* ^2^ = 0.9934	*R* ^2^ = 0.999	*R* ^2^ = 0.9609
*χ* ^2^ = −0.00054	*χ* ^2^ = 11.4896	*χ* ^2^ = 0.2242

### Thermodynamic studies

To investigate the nature of adsorption, thermodynamic parameters viz. enthalpy (Δ*H*°), entropy (Δ*S*°) and Gibbs free energy (ΔG°) were estimated. The following Van’t Hoff equation was used to evaluate thermodynamic parameters:

12 $$ \log \left( {q_{e} /c_{e} } \right) =\Delta H/2.303{\text{RT}} +\Delta S/2.303R $$

**Fig. 4:**
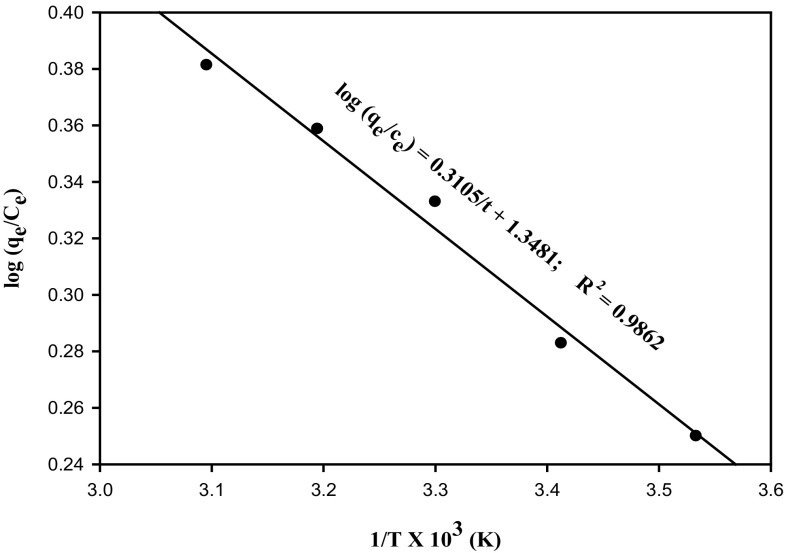
Van’t Hoff plot for adsorption of copper using DNES–CH composite

The negative value of Δ*H* indicates an exothermic reaction; a positive value denotes an endothermic reaction. If the value of Δ*S* is less than zero, it indicates the process is highly reversible and if more than or equal to zero, it indicates the irreversibility of the process. The negative value of Δ*G* indicates the spontaneity of adsorption whereas a positive value of ΔG indicates the non spontaneity of adsorption. Van’t Hoff plot is represented in Fig. 4 and the equation obtained was log (*q*_*e*_/*c*_*e*_) = 0.3105(1/*T*) + 1.3481. The positive experimental Δ*H*° value indicates that the adsorption process was endothermic in nature and there was a possible strong bonding between the metal ion and the adsorbent. As Δ*S*° was more than zero, the adsorption process appears to be irreversible (Desorption studies were hence not attempted). The Gibbs free energy is negative, suggesting the spontaneous nature of the adsorption process. The free energy change (Δ*G*°) increased with increase in temperature (10–50 °C), presumably due to activation of more sites on the surface of the adsorbent (Sengil and Ozacar 2008). The thermodynamic parameters at different temperatures are given in Table 3.

**Table 3:** Thermodynamic parameters calculated by Van’t Hoff equation

Temperature (°K)	∆*G*° (kJ/mol)	∆*H*° (kJ/mol)	∆*S*° (kJ/mol K)
283	−7298.9075	5.9451	25.8122
293	−7557.0295		
303	−7815.1515		
313	−8073.2735		
323	−8331.3955		

The maximum adsorption capacities of a few common adsorbents for copper are shown in Table 4. When compared to the synthetic adsorbents, the maximum adsorption capacity of the natural DNES–CH composite is low. But the advantages of the composite are good enough in minimizing sludge generation and the recovery of the metal from the effluents.

**Table 4:** Comparison of the adsorption capacities (*q*_max_) of different adsorbents

Adsorbent	*q*_max_ (mg/g)	References
*Pseudomonas aeruginosa*	5.83	Tuzen et al. (2008)
Saw dust	4.9	Grimm et al. (2008)
Wheat bran	8.62	Wang et al. (2009)
Chitosan supported on porous glass beads	7.27	Shen et al. (2013)
n-HAp, n-HAp/chitin (n-HApC) composite and n-HAp/chitosan (n-HApCs) composite	4.7, 5.4 and 6.2	Rajiv gandhi et al. (2011)
Immobilization of a synthesized (3-(3-(methoxycarbonyl)benzylidine)hydrazinyl)benzoic acid onto mesoporus silica monoliths	145.98	Awual et al. (2013)
5-Tert-butyl-2-hydroxybenzaldehyde thiosemicarbazone immobilized on to the mesoporus silica	176.27	Awual (2015)
DNES–CH composite	48.3	Present study

### Statistical analysis

Experimentally, highest adsorption capacity was obtained at an adsorbent dosage of 8 g/L, initial metal ion concentration 20 mg/L, and pH 6. With these preliminary results, further experiments on the factors affecting adsorption were carried out using three level full factorial design under RSM with varying levels of adsorbent dosage, metal ion concentration and pH.

The coded and the actual values of the test variables are as given in Table 1. Multiple regression analysis of the data for adsorption yielded the following regression equation.

13 $$ Y = + 95.25 - 1.74 \, X_{1} + 0.56 \, X_{2} - 0.089 \, X_{3} - 5.84 \, X_{1} X_{2} - 2.09 \, X_{1} X_{3} - 1.00 \, X_{2} X_{3} - 16.11 \, X_{1}^{2} - 2.29 \, X_{2}^{2} - 0.82 \, X_{3}^{2} $$

where *Y* is the % adsorption of copper, *X*_1_ adsorbent dosage, *X*_2_ is the metal ion concentration and *X*_3_ is the pH. Solving the regression equation using fmincon function, the optimum set of values for the three variable *X*_1_, *X*_2_ and *X*_3_ were −0.0960, +0.2651 and −0.0936. Hence, it was inferred that for maximum adsorption of copper with the DNES-CH composite, the adsorbent dosage, metal ion concentration and pH should ideally be kept at 7.90 g/L, 20.2651 mg/L and 5.906 respectively.

### Interpretation of the regression analysis

Statistical testing of the model by the Fischer’s statistical test for analysis of variance (ANOVA) yielded a *F*-value of 46.25 with a low *p* value (*p* < 0.0001) indicating the significance of the model (Table 5). Determination coefficient nearer to unity (*R* ^2^ = 0.9585) indicates that only 4.15 % of the total variations are not explained by the model. The predicted *R* ^2^ of 0.8977 is in reasonable agreement with the adjusted *R* ^2^ of 0.9378 (difference is less than 0.2). The results of the full factorial design are represented in Table 6 and it is inferred that the quadratic model is the best fit for the observed biosorption of copper by DNES–CH composite.

**Table 5:** Regression coefficients for adsorption of copper by DNES–CH composite

Model term	Coefficient estimate	Standard error	*F* value	*p* value
Intercept	95.25	1.06	46.25	<0.0001
*X*_1_	−1.74	0.55	9.87	0.0056
*X*_2_	0.56	0.55	1.03	0.3240
*X*_3_	−0.089	0.55	0.026	0.8739
*X*_1_ *X*_2_	−5.84	0.68	74.48	<0.0001
*X*_1_ *X*_3_	−2.09	0.68	9.59	0.0062
*X*_2_ *X*_3_	−1.00	0.68	2.19	0.1563
*X*_1_^2^	−16.11	0.93	301.46	<0.0001
*X*_2_^2^	−2.29	0.93	6.11	0.0236
*X*_3_^2^	−0.82	0.93	0.78	0.3898

All the linear, interaction and squared terms are significant (*p* < 0.5), excluding linear *X*_3_

**Table 6:** Results from full factorial design for copper adsorption by DNES–CH composite

Source of variation	Sum of squares	*df*	Mean square	*F* value	*p* > *F*
Model	2285.25	9	253.92	46.25	<0.0001
Lack of fit	98.82	17	5.81		
Pure error	0.000	1	0.000		
Total	2384.08	27			

### Interaction effects of biosorption variables

**Fig. 5:**
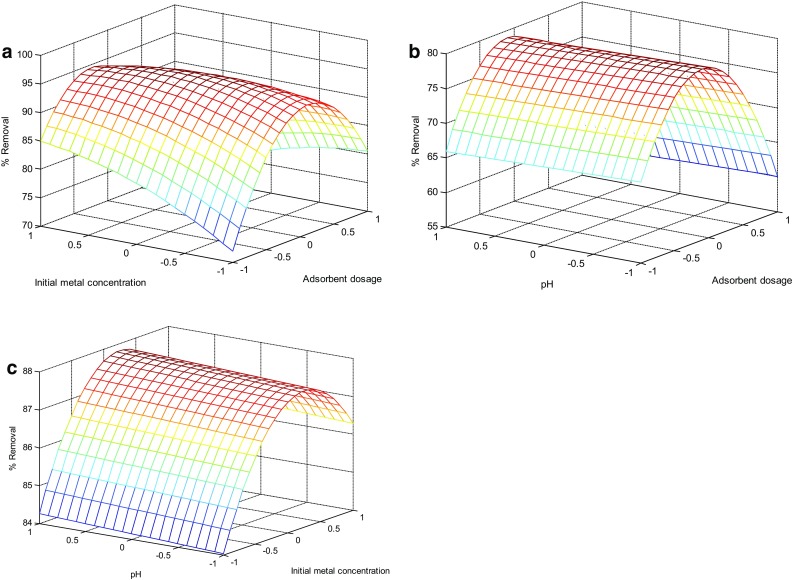
**a** Surface plot for the effects of adsorbent dosage and initial metal concentration of copper on % removal. **b** Surface plot for the effects of adsorbent dosage and pH of copper on % removal. **c** Surface plot for the effects of pH and initial metal concentration of copper on % removal

To know the interactive effects of biosorption variables, surface plots drawn using Matlab 2008 are provided in Fig. 5a–c. From the curvature of the contour, it was identified that the biosorpiton of copper is strongly influenced by the adsorbent dosage, initial metal concentration and pH.

### SEM–EDS analysis

**Fig. 6:**
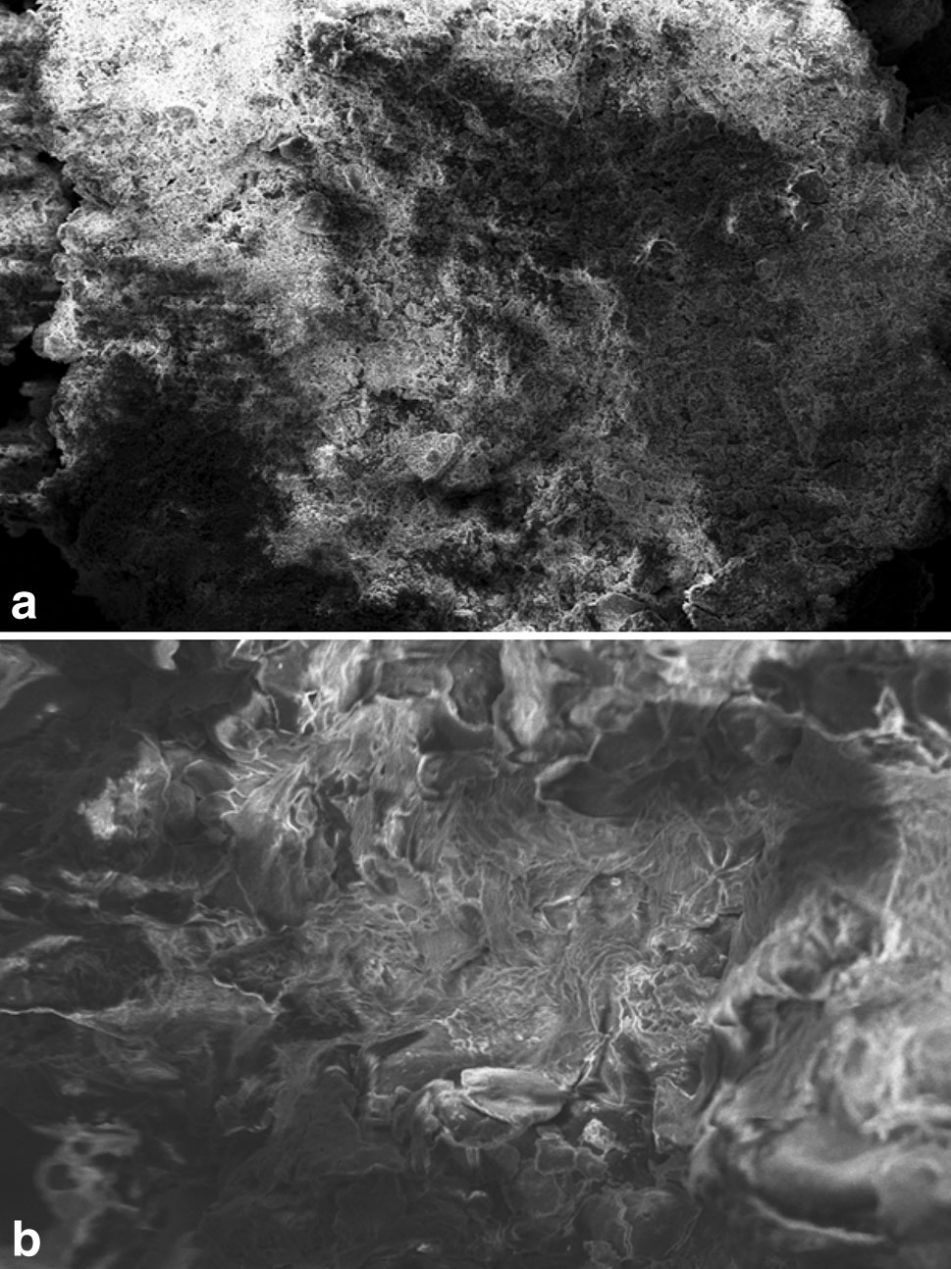
**a** SEM micrograph of native DNES–CH composite. **b** SEM micrograph of DNES–CH composite after adsorption of copper

**Fig. 7:**
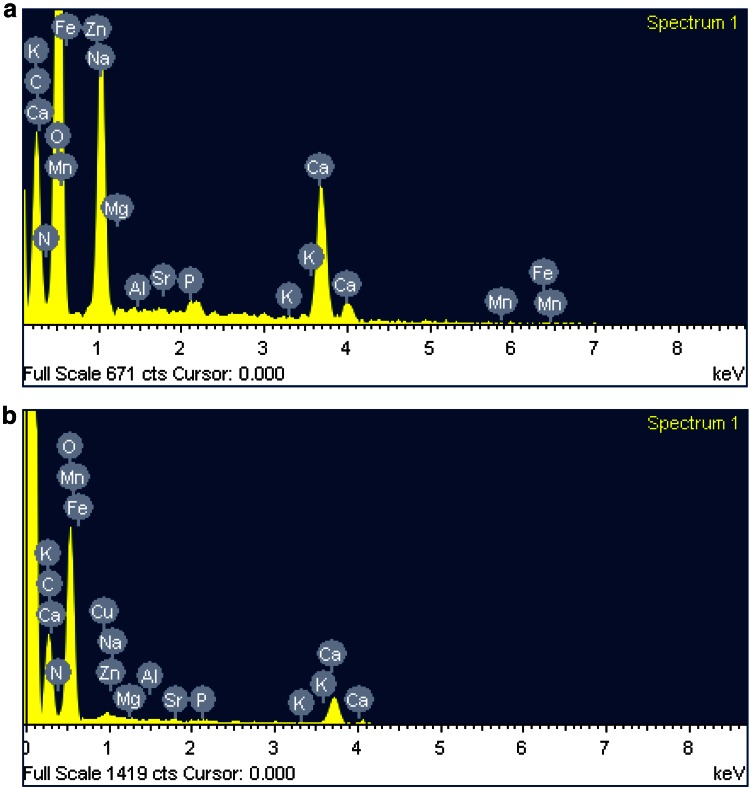
**a** EDS micrograph of native DNES–CH composite. **b** EDS micrograph of DNES–CH composite after adsorption of copper

SEM analyses of the biosorbent material, DNES–CH composite, before and after adsorption of copper are presented in Fig. 6a, b, respectively. After adsorption of copper, the surface morphology of the adsorbent changed to rough, more uneven and heterogeneous nature which could probably be due to the reorganization of surface functional groups binding the metal ions. From the EDS analysis before biosorption, the major constituent of the eggshells—CaCO_3_, along with the elements *N* (chitosan provides amino groups), P and the metals Mg, Na, K, Mn, Zn, Fe, Al, Sr etc. is evident to be present in the composite (Fig. 7a). After adsorption of copper (Fig. 7b), the EDS analysis showed the presence of copper ions (3.71 %) with a concomitant decrease in the percentages of Mg, Na and Zn, suggesting that the adsorption of copper by the composite may also include the ion-exchange mechanism.

### Adsorption mechanism through FTIR spectral analysis

**Fig. 8:**
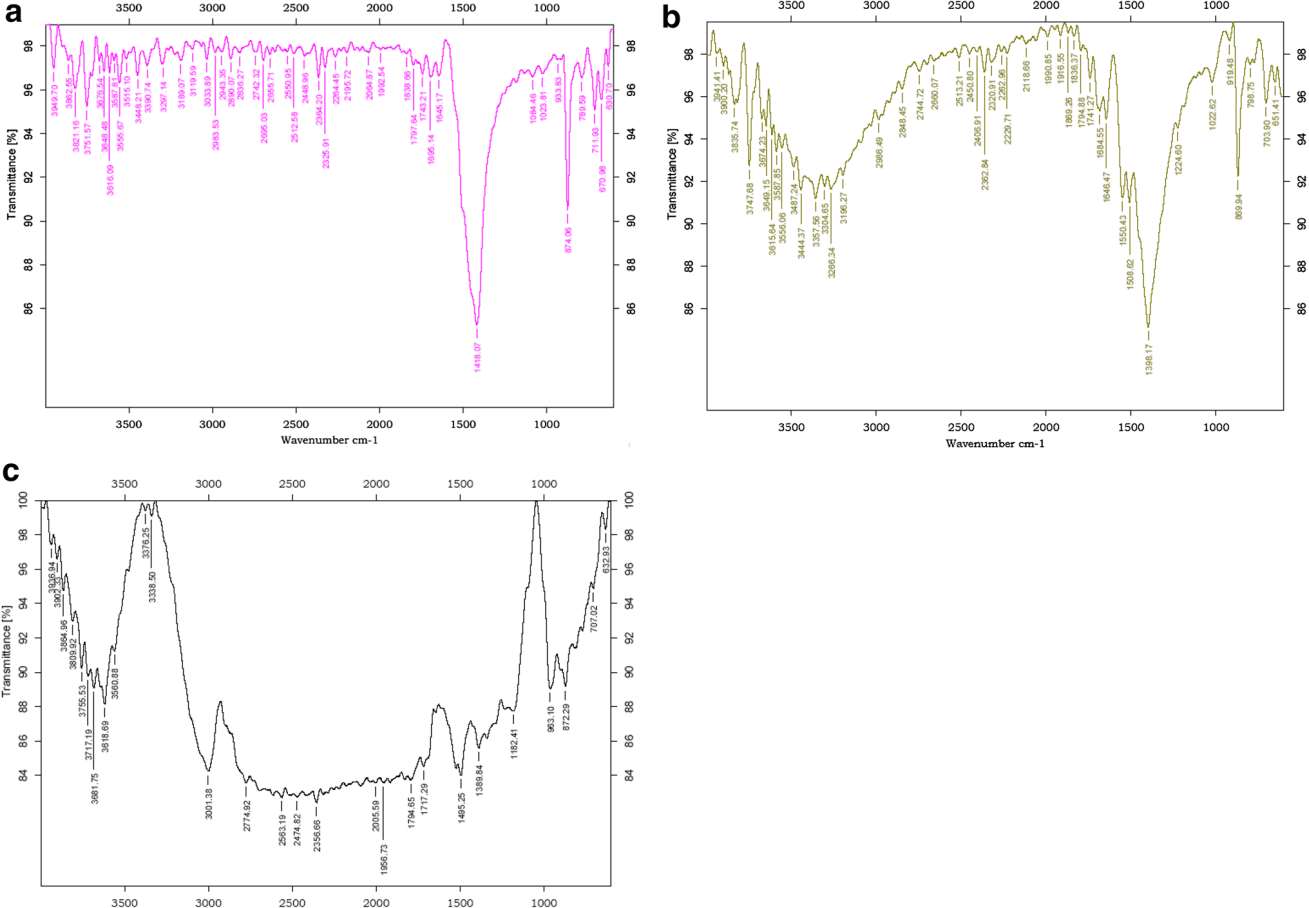
**a** FTIR spectrum of native DNES. **b** FTIR spectrum of native DNES–CH composite. **c** FTIR spectrum of copper loaded DNES–CH composite

The adsorption mechanism was investigated using FTIR analysis of the native DNES, native and the metal loaded DNES-CH composite in the range of 400–4000 cm^−1^ (Fig. 8a–c). The native DNES FTIR spectra showed sharp peaks at 874, 1418 cm^−1^ but after forming composite with chitosan, provided a number of functional groups which correspond to the amino and the carboxyl. In DNES-CH composite, ES is porous in nature and provides an efficient and strong support for the CH in forming the scaffold with more number of hydroxyl, carboxyl, amino and alcohol groups available to chelate the copper metal ions. FTIR spectra showed marked differences before and after adsorption of copper. Broadly, the spectrum could be divided into three distinct regions viz. 630–1000, 1180–3000, and 3300–3700 cm^−1^ wherein there are significant variations in the absorption patterns and the groups assigned for biosorption as given in Table 7.

**Table 7:** FTIR peaks and their corresponding groups

DNES–CH composite spectrum peaks (λ)	Copper treated DNES–CH composite, spectrum peaks (*λ*)	Assignment
3747	3755, 3717	O–H & N–H groups
3357	3376	
3304	3338	
2986	3001	Carboxyl groups
1741	1717	Amines
1550	1508	N–H bending vibration
1398	1389	C-N, Amines
1224	1182	
1022	963	
869	872	NH_3_ rocking vibration
651	632	Cu–O stretching

After interaction with the copper ions, it was identified that the sharp band at 3747 split into 3755 and 3717 cm^−1^ indicating that both O and N atoms present on the adsorbent surface played a significant role in binding the metal ions (Table 7); the bands at 3357 and 3304 cm^−1^ were assigned to O–H & N–H, and inter hydrogen bonds’ vibration shifted to 3376 and 3338 cm^−1^. A shift of band at 2986 cm^−1^ to a broad wide band at 3001 cm^−1^ indicates the involvement of carboxyl groups. After exposure to copper, the bands corresponding to amine shifted from 1741 to 1717 cm^−1^ and the bands at 1684, 1646 cm^−1^ disappeared. Moreover, the adsorption intensity for the N–H bending vibration at 1550 and 1508 cm^−1^ shifted to 1495 cm^−1^ and decreased after copper uptake. This observation is supported by an extreme change in absorption intensity for C–N stretching vibrations at 1398–1389 cm^−1^. The metal ion uptake has led to a significant change in the absorption intensity of NH_3_ rocking vibration from 869 to 872 cm^−1^. Variations in the absorption peaks at 630–700 cm^−1^ in the two samples (before and after treatment with copper) are indicative of Cu–O stretching in the copper loaded DNES-CH (Vinod and Miroslav 2013). In general, the adsorption capacity depends upon the porosity as well as functional groups present on the adsorbent surface (Kumar et al. 2010). Accordingly, the observed changes in the adsorption intensity and the wave number of functional groups strongly suggest the occurrence of complexation between N and O atoms on the adsorbent binding sites and the copper ions. The FTIR analysis suggests that the maximum adsorption of copper by the DNES–CH composite is due to the availability of more number of N and O atoms.

### X-ray diffraction (XRD) analysis

**Fig. 9:**
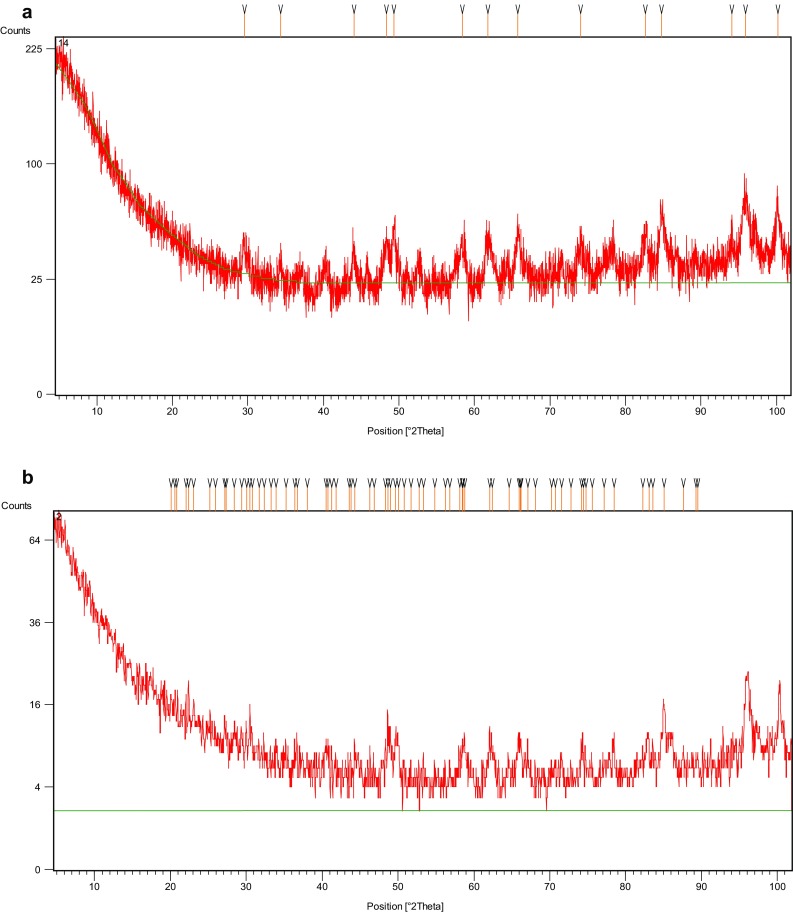
**a** XRD of native DNES–CH composite. **b** XRD of DNES–CH composite treated with copper

The native DNES–CH X-ray diffractogram (as shown in Fig. 9a) exhibited sharp peaks at 2*θ* positions 29°, 34°, 44°, 48°, 49°, 58°, 61°, 65°, 74°, 82°, 84°, 94°, 95° and 100°. X-ray diffractogram analysis of the copper loaded DNES–CH composite (as shown in Fig. 9b) showed the amorphous nature, with sharp peaks at 2*θ* positions of 20°, 22°, 23°, 25°, 27°, 28°, 30°, 48° and 85°. In addition, the peaks at 43.7°, 50° and 74.2° (JCPDS copper: 04-0836) suggest the presence of copper while the peaks at 2*θ* positions of 32.3°, 35.2°, 38°, 46.2°, 48.6°, 51.7°, 53.4°, 56.8°, 58.1°, 65.9°, 66.1°, 68.0°, 71.5°, 72.7°, 74.7°, 82.2°, 83.0°, 83.6°, 87.6° and 89.5° (JCPDS CuO: 80-1916) indicate the formation of CuO by the reaction of the metal with the hydroxyl and the carboxyl groups present on the adsorbent. The said reaction may be due to the electron pair sharing between the copper metal and the reactive carboxyl/hydroxyl groups in the DNES–CH composite.

## Conclusions

Initial experiments following the methodology of ‘one variable at a time’ were conducted for adsorption of copper by varying the factors: contact time, temperature, adsorbate pH, adsorbate concentration, adsorbent size and adsorbent dosage. From the experimental data, the equilibrium was found to be attained at 60 min of contact time due to the presence of more and/or active functional groups. The percentage removal of copper decreased with the increase in the initial concentration of the adsorbate. The copper removal increased rapidly with the increasing adsorbent dosage initially, and attained equilibrium at 8 g/L. Percentage removal of copper from aqueous solution increased significantly with the increase in pH from 4 to 6; thereafter further increase in pH decreased the percentage removal. In a nut shell, the results of the present study demonstrate that DNES-CH composite could be considered for the effective and economic treatment of the industrial waste water containing copper, and achieve levels well below the permissible limit of 1.3 mg/L of copper suggested for safe drinking water.

## Abbreviations

DNES: *Dromaius novaehollandiae* eggshell
CH: Chitosan
AAS: Atomic absorption spectrophotometer
SEM: Scanning electron microscopy
FTIR: Fourier transform infrared spectroscopy
XRD: X-ray diffractometer
